# Exploring the influencing factors on acne, melasma, and rosacea: A case–control study in China

**DOI:** 10.1111/jocd.16499

**Published:** 2024-08-02

**Authors:** Qingyue Xia, Zhaopeng Wang, Yingdan Tang, Xingbao Luan, Tianxurun Deng, Lipan Fan, Hongjin Wu, Yuan Li, Xiaomei Cui, Yang Zhao, Dan Luo

**Affiliations:** ^1^ Department of Dermatology The First Affiliated Hospital of Nanjing Medical University Nanjing China; ^2^ Department of Dermatology China‐Japan Friendship Hospital Beijing China; ^3^ Department of Biostatistics School of Public Health, Nanjing Medical University Nanjing China; ^4^ Department of Dermatology Children's Hospital of Nanjing Medical University Nanjing China

**Keywords:** acne, case–control, influencing factor, melasma, rosacea

## Abstract

**Background:**

The severity and treatment response of acne, melasma, and rosacea may be influenced by various currently unclear internal and external factors. This study aimed to provide evidence to the influencing factors for the mentioned conditions through a real‐world case–control study.

**Methods:**

An online survey consisting of 60 questions was implemented, collecting information on demographics, socioeconomics, genetic factors, lifestyle habits, environmental exposures, and skin care behaviors. Then we constructed univariate and multivariate logistic regressions. Furthermore, we analyzed the dose–response relationship between exposure and outcome.

**Results:**

A total of 399 individuals, including 94 acne patients, 107 melasma patients, and 91 rosacea patients were included. Acne and melasma were positively correlated with screen time (acne: odds ratio [OR]: 2.24, 95% confidence interval [CI]: 1.25–4.02; melasma: OR: 1.59, 95% CI: 1.09–2.31), while exercise exerted a protective effect on both acne (OR: 0.31, 95% CI: 0.13–0.77) and melasma (OR: 0.42, 95% CI: 0.22–0.80) in a dose–response relationship. In addition, males were associated with an elevated risk of acne (OR: 6.62, 95% CI: 1.01–43.26). Aging (OR: 1.15, 95% CI: 1.07–1.24) and irregular bowel movements (OR: 2.99, 95% CI: 1.11–8.08) were independent risk factors for melasma. Rosacea was positively associated with BMI (OR: 1.17, 95% CI: 1.01–1.35).

**Conclusion:**

In our study, we highlighted exercise as an independent protective factor for both acne and melasma in a dose–response trend. Inversely, extended use of electronic equipment was independently associated with higher risks of acne and melasma. Rosacea, however, was more likely to be related with BMI.

## 1 INTRODUCTION

Facial disfiguring skin diseases refer to a group of skin conditions that primarily affect the facial and other exposed skin areas, causing varying degrees of impact on patients' appearance, social interactions, and psychological well‐being. Acne, melasma, and rosacea, which often affect the face and manifest as pigmentation, papules, pustules, and scarring on the skin, are among the common disfiguring skin diseases. All of them, especially acne, was acknowledged the top 10 skin diseases across high‐ and low‐income countries in the Global Burden of Skin Disease study in 2010.^1^ Thus, more prevention and treatment strategies of facial disfiguring skin diseases need to be updated. Etiological data can provide new directions for prevention and treatment and is therefore an area of urgent research.

The concept of “exposome” refers to the cumulative sum of external and internal environmental exposures that an individual experiences from conception to death.^2^ In 2017, Krutmann et al.^3^ first evaluated the impact of exposures on photoaging. Subsequently, Dreno et al.^4^ explored the key exposure factors associated with acne in several countries in 2019, including France, Germany, Italy, Brazil, Canada, and Russia, using a questionnaire survey. They found that nutrition, pollution, stress, rough skincare practices, climate, and sunlight exposure were the most common factors considered to be associated with acne. However, research on relevant exposure factors in Asian populations, especially in the Chinese population, is still lacking. Melasma is an acquired pigmentary disorder that primarily affects sun‐exposed areas, commonly occurring in women of childbearing age. While sunlight exposure, pregnancy, sex hormones, cosmetics, skin inflammation processes, photosensitizing drugs, and genetic susceptibility are known triggering factors,^5^ the precise pathophysiological mechanisms underlying melasma are not fully understood. There is a lack of epidemiological case–control studies on melasma, and to date, there has been limited research systematically investigating its exposure factors. Rosacea is a common chronic inflammatory skin disease characterized by a “flushing” facial appearance. Its pathophysiological mechanisms are not fully understood but involve complex interactions among genetics, ultraviolet radiation, microorganisms, impaired skin barrier, neurovascular dysfunction, and immune system dysregulation.^6^ However, there is a lack of research on the relevant exposure factors for rosacea.

Therefore, accurate report of the risk factors of the above diseases can provide effective clues for their occurrence and development, and solid evidence for their prevention and treatment. This study aims to explore the common internal and external exposure factors associated with acne, melasma, and rosacea through real‐world, case–control research.

## 2 METHODS

A case–control study was conducted between August 2023 and May 2024, involving patients with facial skin diseases and healthy controls from the Hello Cloud Doctor platform, which is an Internet‐based, post‐diagnosis education platform with dermatology patients, and health consultants from different regions in China. The case group consisted of patients clinically diagnosed with facial acne, melasma, or rosacea by professional dermatologists, while the control group comprised healthy individuals without present diseases. The ratio of cases to controls was at least maintained at 1:1 in both groups to ensure adequate statistical power. The exclusion criteria for both case and control groups were as following: (1) Refuse to sign informed consent, (2) Insufficient validity or incompleteness of questionnaire completion, (3) Pregnant or lactating women, (4) Comorbidity that may influence the outcomes (e.g., malignant tumors, polycystic ovary syndrome and chronic liver disease).

**FIGURE 1:**
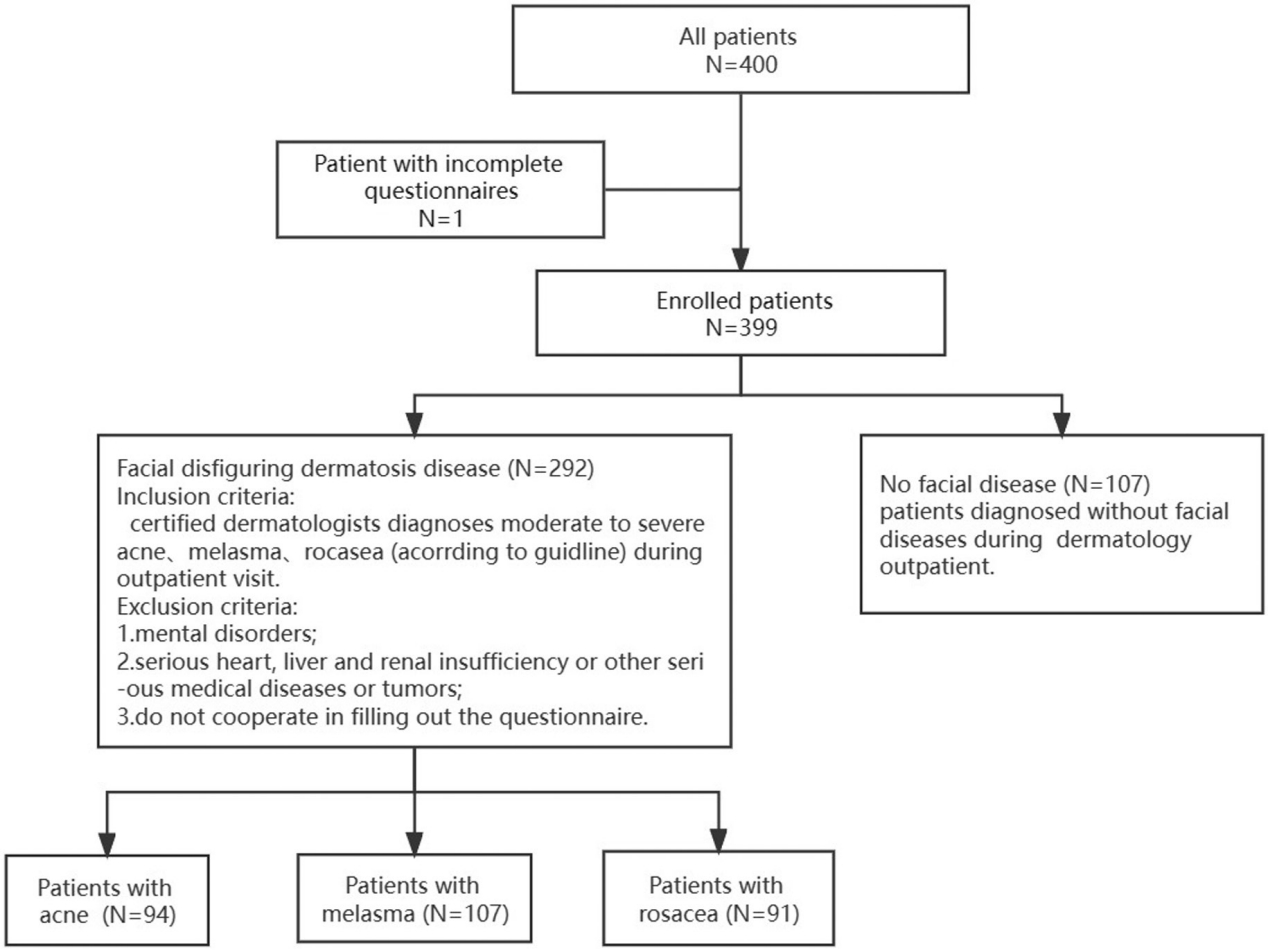
Study flow chart.

The survey was conducted through the Hello Medical Internet Service Cloud platform, where the purpose of the survey was explained. Informed consent was obtained from all participants at the beginning of the questionnaire. The medical ethics committee approved this study (for detailed information, please refer to the Ethical statements). The questionnaire consisted of 60 questions, covering demographic and socioeconomic data, lifestyle habits, environmental exposures, genetic factors, digital device usage, skin condition and skin care habits, and the impact of skin diseases on daily life. Participants completed the survey by recalling and filling in the information. The flowchart of the survey process is shown in Figure 1.

Continuous variables were described as mean ± SD or median (interquartile range, IQR) when data did not follow a normal distribution, and the categorical variables were expressed as frequency (proportion). The presence of normal distribution was verified by Shapiro–Wilk test.^7^

The exposure factors in the questionnaire were evaluated as potential factors influencing disfiguring dermatosis (acne, melasma, and rosacea) in all participants using univariate logistic regressions. Any factors with *p* < 0.2 in univariate analyses were included in multivariable logistic regressions.^8^ The univariate and multivariable logistic regressions all adjusted for sex, age, and nation. In order to further explore the potential pattern of the continuous variables on the disfiguring dermatosis, we divided the continuous variables into ordered categorical variables according to the questionnaire information, and performed categorical analysis with the lowest frequency as the reference group to evaluate the dose–response relationship between factors and disease.^9^ The *p*‐value for the trend is verified by the Cochran Armitage test.^10^, ^11^ Odds ratios (ORs) with 95% confidence intervals (CIs) were presented.

All data analyses were performed using SAS version 9.4 (SAS Institute Inc)^12^ and figures were created using R software, version 4.0.0 (R Foundation for Statistical Computing, Vienna, Austria).^13^ All reported *p*‐values are two‐sided at a type I error rate of 0.05, and *p* < 0.05 was considered statistically significant.

## 3 RESULTS

A total of 399 individuals were deemed suitable for statistical analysis: the disfiguring dermatosis group comprised 292 individuals (94(32.19%) individuals with acne, 107(36.64%) individuals with melasma, and 91(31.16%) individuals with rosacea), while the control group consisted of 107 individuals. All individuals were Asians with skin color ranging from Fitzpatrick II to V. The disfiguring dermatosis group and the control group exhibited similar median age, gender distribution, and national distribution. However, participants with disfiguring dermatosis had a significantly higher body mass index (BMI) of 20.96 compared to those without any dermatosis (20.52). Among the disease subgroups, patients with melasma (33.00) had a significantly higher age compared to the control group (27.59). The proportion of male acne patients was higher than that of the control group, as was the proportion of female melasma patients. Both acne (21.47) and melasma (21.3) patients had significantly higher BMIs than the control group (20.52) (Table 1). For more detailed information on patient baseline characteristics refer to Table S1.

**TABLE 1:** Brief information on baseline characters of Facial disfiguring dermatosis, acne, melasma, rosacea, and healthy controls.

Baseline characteristic	No disease (*N* = 107)	Disfiguring dermatosis (*N* = 292)	Acne (*N* = 94)	Melasma (*N* = 107)	Rosacea (*N* = 91)
Demographic and socioeconomic characteristics					
Sex					
Female	91 (85.05)	245 (83.90)	73 (77.66)	97 (90.65)	75 (82.42)
Male	16 (14.95)	47 (16.10)	21 (22.34)	10 (9.35)	16 (17.58)
Age	27.59 (8.8)	29.57 (11.00)	26.33 (10.00)	33.00 (8.54)	28.00 (10.00)
Nation					
Han nationality	104 (97.20)	278 (95.21)	89 (94.68)	100 (93.46)	89 (97.80)
Other	3 (2.80)	14 (4.79)	5 (5.32)	7 (6.54)	2 (2.20)
BMI	20.52 (2.62)	20.96 (3.94)	21.47 (4.89)	21.30 (3.48)	20.58 (3.61)

Abbreviation: BMI, body mass index.

### 3.1 Influencing factors of facial disfiguring dermatosis, acne, melasma, and rosacea

**FIGURE 2:**
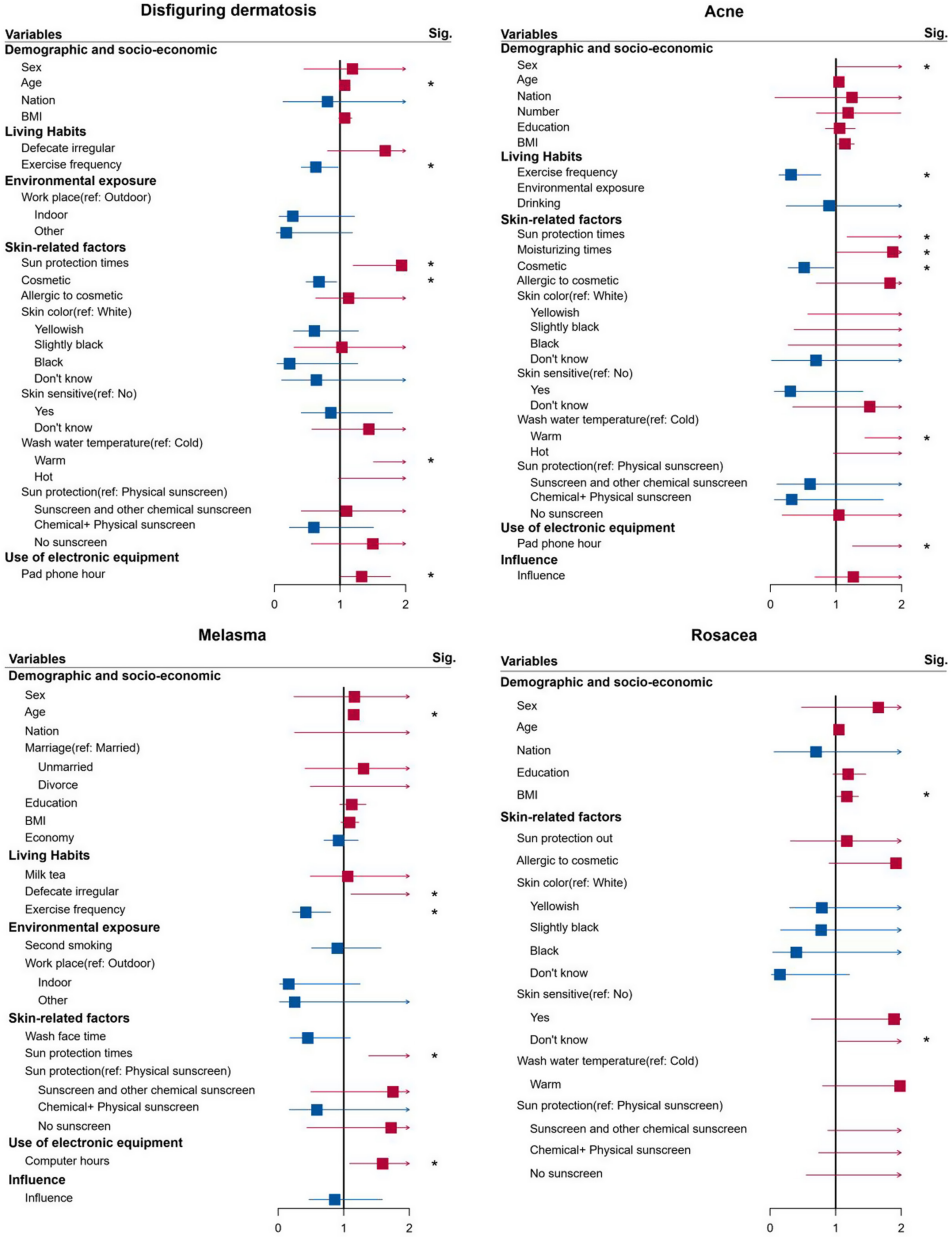
The association of influencing factors with facial disfiguring dermatosis, acne, melisma, and rosacea. The results of associations from the multi‐variate logistic regression. Estimates are odds ratios (ORs) and 95% CI. The model included all risk factors with *p <* 0.2 obtained by univariate regression, adjusting for age, sex, and nation. Significance (Sig.): *p ≤ 0.05. Blue represents OR <1; red represents OR >1. BMI, body mass index.

The investigation of any potential risk factors and protective factors influencing disfiguring dermatosis involved the utilization of univariate and multivariate logistic regression analyses. Given that previous research has indicated the influence of age, gender, and ethnicity on diseases such as acne,^14^ these variables were considered as covariates in the analysis. The results of univariate and multivariate logistic regression analyses were presented in Table 2, Tables S2–S5, and Figure 2.

**TABLE 2:** Association of exposure factors with facial disfiguring dermatosis, acne, melasma, and rosacea using univariate analyses.

Variables	Disfiguring dermatosis		Acne		Melasma		Rosacea	
	OR (95% CI)	*p*‐value	OR (95% CI)	*p*‐value	OR (95% CI)	*p*‐value	OR (95% CI)	*p*‐value
Demographic and socioeconomic characteristics								
Sex	1.15 (0.62, 2.15)	0.6548	1.55 (0.75, 3.22)	0.2388	0.74 (0.31, 1.76)	0.4925	1.20 (0.56, 2.57)	0.6464
Age	1.02 (0.99, 1.05)	0.2853	0.98 (0.94, 1.02)	0.2545	1.07 (1.03, 1.11)	0.0007	1.00 (0.96, 1.04)	0.8741
Nation	1.70 (0.48, 6.06)	0.4125	2.06 (0.48, 8.89)	0.3330	2.54 (0.60, 10.77)	0.2051	0.80 (0.13, 4.91)	0.8059
Marriage								
Married	Ref.		Ref.		Ref.		Ref.	
Unmarried	1.22 (0.69, 2.18)	0.4938	1.08 (0.49, 2.34)	0.8545	0.99 (0.49, 2.01)	0.9750	1.32 (0.63, 2.77)	0.4599
Divorce	3.84 (0.48, 30.77)	0.2056	1.64 (0.10, 27.18)	0.7293	5.06 (0.58, 43.93)	0.1413	3.97 (0.40, 39.75)	0.2404
Widowed	545544 (0.00, I)	0.9895	4.79E6 (0.00, I)	0.9858				
Family member	0.96 (0.77, 1.20)	0.7219	0.80 (0.60, 1.07)	0.1379	1.03 (0.78, 1.36)	0.8400	1.00 (0.75, 1.34)	0.9941
Education	1.02 (0.94, 1.10)	0.6886	1.08 (0.97, 1.20)	0.1634	0.93 (0.84, 1.02)	0.1324	1.10 (0.99, 1.23)	0.0839
BMI	1.10 (1.03, 1.17)	0.0037	1.12 (1.04, 1.21)	0.0019	1.13 (1.03, 1.23)	0.0078	1.07 (1.00, 1.15)	0.0676
Economy	0.94 (0.81, 1.08)	0.3495	0.97 (0.81, 1.15)	0.6954	0.88 (0.73, 1.05)	0.1463	0.99 (0.83, 1.18)	0.9145
Living habits								
Milk habbit	0.97 (0.62, 1.53)	0.9003	1.00 (0.57, 1.76)	0.9937	1.10 (0.62, 1.93)	0.7505	0.83 (0.47, 1.47)	0.5158
Milk content	0.92 (0.69, 1.23)	0.5821	0.99 (0.69, 1.41)	0.9463	0.97 (0.68, 1.40)	0.8785	0.79 (0.54, 1.16)	0.2318
Soft drink	1.02 (0.72, 1.44)	0.9297	0.79 (0.50, 1.26)	0.3184	1.29 (0.84, 1.98)	0.2456	0.94 (0.60, 1.48)	0.7914
Milk tea	1.08 (0.75, 1.55)	0.6795	1.00 (0.63, 1.57)	0.9899	1.52 (0.96, 2.41)	0.0740	0.76 (0.47, 1.24)	0.2726
Juice	1.21 (0.84, 1.75)	0.2944	1.06 (0.67, 1.70)	0.7970	1.29 (0.83, 2.02)	0.2627	1.20 (0.77, 1.86)	0.4259
Diarrhea	1.10 (0.87, 1.38)	0.4419	1.02 (0.76, 1.37)	0.8909	1.13 (0.85, 1.49)	0.3983	1.06 (0.77, 1.44)	0.7337
Defecate times	1.21 (0.88, 1.67)	0.2339	1.14 (0.76, 1.72)	0.5218	1.18 (0.80, 1.74)	0.4058	1.29 (0.87, 1.93)	0.2081
Irregular defecation	1.57 (0.90, 2.73)	0.1093	1.37 (0.69, 2.72)	0.3718	2.51 (1.30, 4.83)	0.0060	0.98 (0.48, 2.02)	0.9566
Pet	1.53 (0.76, 3.11)	0.2376	1.41 (0.58, 3.42)	0.4496	1.68 (0.72, 3.91)	0.2281	1.72 (0.74, 3.97)	0.2062
Exercise frequency	0.65 (0.49, 0.88)	0.0044	0.56 (0.37, 0.85)	0.0061	0.48 (0.32, 0.73)	0.0006	0.85 (0.58, 1.24)	0.3938
Sedentariness	0.97 (0.79, 1.19)	0.7512	0.93 (0.72, 1.22)	0.6152	0.95 (0.75, 1.20)	0.6525	1.10 (0.85, 1.44)	0.4743
Genetic factor								
First‐degree relatives	1.04 (0.65, 1.68)	0.8637	1.34 (0.74, 2.43)	0.3324	0.89 (0.48, 1.62)	0.6940	0.85 (0.46, 1.57)	0.6045
Second‐degree relative	0.80 (0.48, 1.34)	0.4012	0.78 (0.41, 1.51)	0.4638	0.74 (0.39, 1.42)	0.3663	0.90 (0.48, 1.70)	0.7461
Blood								
Type A	Ref.		Ref.		Ref.		Ref.	
Type B	0.98 (0.48, 1.97)	0.9467	0.67 (0.26, 1.69)	0.3936	0.91 (0.38, 2.19)	0.8293	1.51 (0.61, 3.73)	0.3739
Type O	1.16 (0.59, 2.28)	0.6659	0.99 (0.43, 2.32)	0.9896	1.01 (0.43, 2.35)	0.9826	1.52 (0.63, 3.62)	0.3493
Type AB	1.11 (0.40, 3.06)	0.8407	0.65 (0.16, 2.65)	0.5499	1.36 (0.40, 4.58)	0.6229	1.27 (0.35, 4.69)	0.7173
Don't know	1.25 (0.62, 2.50)	0.5345	1.33 (0.57, 3.09)	0.5114	1.21 (0.50, 2.94)	0.6721	1.12 (0.44, 2.82)	0.8118
Environmental exposure								
Smoking	1.23 (0.54, 2.81)	0.6155	1.24 (0.47, 3.28)	0.6599	0.99 (0.34, 2.91)	0.9896	1.46 (0.53, 4.07)	0.4642
Smoking year	1.02 (0.74, 1.40)	0.9223	0.93 (0.62, 1.40)	0.7268	0.96 (0.64, 1.44)	0.8449	1.16 (0.80, 1.69)	0.4388
Smoking frequency	1.16 (0.61, 2.20)	0.6430	1.05 (0.47, 2.35)	0.9013	1.16 (0.54, 2.49)	0.7014	1.28 (0.56, 2.90)	0.5554
Second smoking	1.16 (0.87, 1.54)	0.3113	1.11 (0.79, 1.56)	0.5579	1.34 (0.96, 1.88)	0.0892	0.95 (0.65, 1.39)	0.7989
Drinking	1.47 (0.79, 2.70)	0.2214	1.67 (0.81, 3.45)	0.1637	1.15 (0.53, 2.48)	0.7295	1.60 (0.74, 3.44)	0.2302
Drinking year	1.10 (0.88, 1.37)	0.3986	1.14 (0.87, 1.48)	0.3384	1.02 (0.78, 1.33)	0.8917	1.15 (0.88, 1.51)	0.2946
Drinking frequency	1.09 (0.82, 1.47)	0.5490	1.17 (0.83, 1.63)	0.3734	1.00 (0.69, 1.45)	0.9918	1.13 (0.79, 1.62)	0.5146
Workplace								
Outdoor	Ref.		Ref.		Ref.		Ref.	
Indoor	0.34 (0.10, 1.17)	0.0871	0.45 (0.11, 1.89)	0.2765	0.24 (0.06, 0.97)	0.0445	0.43 (0.10, 1.77)	0.2413
Other	0.45 (0.10, 2.07)	0.3063	0.79 (0.14, 4.57)	0.7964	0.31 (0.05, 1.90)	0.2071	0.34 (0.05, 2.25)	0.2634
Sun shower	1.22 (0.85, 1.77)	0.2801	1.11 (0.70, 1.76)	0.6496	1.29 (0.85, 1.96)	0.2380	1.16 (0.74, 1.82)	0.5287
Skin‐related factors								
Skin color								
White	Ref.		Ref.		Ref.		Ref.	
Yellowish	0.76 (0.45, 1.28)	0.3014	0.78 (0.40, 1.52)	0.4710	0.83 (0.43, 1.60)	0.5781	0.63 (0.33, 1.20)	0.1607
Slightly black	1.16 (0.47, 2.86)	0.7497	1.00 (0.31, 3.22)	0.9991	1.66 (0.58, 4.76)	0.3455	0.95 (0.31, 2.91)	0.9294
Black	0.39 (0.09, 1.59)	0.1886	0.15 (0.01, 1.57)	0.1133	0.68 (0.11, 4.28)	0.6785	0.38 (0.06, 2.39)	0.3055
Don't know	0.89 (0.32, 2.47)	0.8184	1.01 (0.29, 3.50)	0.9854	0.98 (0.26, 3.72)	0.9806	0.56 (0.14, 2.21)	0.4084
Skin sensitive								
No	Ref.		Ref.		Ref.		Ref.	
Yes	1.46 (0.88, 2.41)	0.1392	2.54 (1.29, 5.01)	0.0069	0.87 (0.46, 1.62)	0.6534	1.60 (0.81, 3.16)	0.1746
Don't know	2.19 (1.12, 4.29)	0.0221	3.01 (1.27, 7.16)	0.0127	1.43 (0.64, 3.19)	0.3773	2.38 (1.03, 5.48)	0.0419
Wash water temperature								
Cold	Ref.							
Warm	1.73 (1.09, 2.74)	0.0190	2.14 (1.18, 3.89)	0.0127	1.45 (0.82, 2.56)	0.2029	1.79 (0.99, 3.22)	0.0540
Hot	2.87 (0.61, 13.57)	0.1829	4.92 (0.92, 26.38)	0.0631	0.97 (0.11, 8.72)	0.9788	2.46 (0.39, 15.76)	0.3411
Wash face time	0.77 (0.48, 1.25)	0.2950	0.95 (0.49, 1.84)	0.8877	0.67 (0.39, 1.16)	0.1538	0.81 (0.43, 1.51)	0.5013
Sun protection								
Physical sunscreen	Ref.		Ref.		Ref.		Ref.	
Sunscreen and other chemical sunscreen	1.74 (0.92, 3.28)	0.0876	1.20 (0.55, 2.60)	0.6456	2.49 (1.14, 5.43)	0.0226	1.95 (0.86, 4.42)	0.1111
Chemical+ physical sunscreen	1.07 (0.60, 1.91)	0.8056	0.56 (0.26, 1.19)	0.1317	1.34 (0.64, 2.82)	0.4392	1.58 (0.75, 3.34)	0.2263
No sunscreen	1.16 (0.57, 2.34)	0.6820	0.64 (0.26, 1.57)	0.3270	1.44 (0.56, 3.73)	0.4469	1.68 (0.67, 4.23)	0.2678
Sun protection times	1.31 (0.97, 1.77)	0.0827	1.31 (0.88, 1.94)	0.1781	1.64 (1.12, 2.38)	0.0104	0.97 (0.62, 1.52)	0.8905
Moisturizing times	1.08 (0.87, 1.34)	0.4636	1.22 (0.92, 1.61)	0.1687	1.09 (0.83, 1.43)	0.5448	0.98 (0.73, 1.32)	0.8901
Facial mask	1.04 (0.82, 1.32)	0.7643	0.89 (0.64, 1.23)	0.4876	1.13 (0.84, 1.52)	0.4319	1.12 (0.82, 1.52)	0.4771
Exfoliate	1.04 (0.89, 1.22)	0.5887	1.04 (0.85, 1.26)	0.7253	1.12 (0.93, 1.35)	0.2309	0.95 (0.78, 1.16)	0.6326
Cosmetic	0.84 (0.66, 1.06)	0.1457	0.79 (0.58, 1.07)	0.1285	0.84 (0.63, 1.13)	0.2470	0.86 (0.63, 1.18)	0.3533
Allergic to cosmetic	1.49 (1.04, 2.14)	0.0315	1.90 (1.19, 3.01)	0.0067	1.27 (0.80, 2.01)	0.3112	1.49 (0.94, 2.39)	0.0925
Use of electronic equipment								
Computer hours	1.08 (0.91, 1.28)	0.3815	1.03 (0.83, 1.27)	0.8190	1.26 (1.01, 1.57)	0.0368	1.02 (0.83, 1.26)	0.8312
Pad phone hour	1.16 (0.96, 1.42)	0.1286	1.32 (1.02, 1.71)	0.0339	1.10 (0.86, 1.41)	0.4513	1.10 (0.86, 1.41)	0.4303
Influence on daily life								
Influence	1.01 (0.76, 1.33)	0.9541	1.48 (1.05, 2.08)	0.0253	0.59 (0.40, 0.88)	0.0097	1.05 (0.74, 1.50)	0.7705

*Note*: The results of associations from the univariate logistic regression. Estimates are showed as odds ratios (OR) (95% CI). The univariate logistic models adjusted for age, sex, and nation.

Abbreviation: BMI, body mass index.

The results of the statistical analysis revealed that age, exercise frequency, wash water temperature, frequency of sun protection, cosmetic usage, and duration of pad and phone usage were identified as independent, and significant influencing factors for overall disfiguring dermatosis. Initially, the univariate analysis indicated that individuals who exhibited sensitivity to cosmetics or were uncertain about skin sensitivity were at a higher risk of developing disfiguring dermatosis. However, upon conducting multivariate analysis, these factors no longer demonstrated statistical significance. Furthermore, the multivariate results indicated that older age (OR = 1.06, 95% CI: 1.01–1.11, *p* = 0.0276), washing the face with warm water as opposed to cold water (OR = 3.00, 95% CI: 1.51–5.95, *p* = 0.0016), increased frequency of sun protection (OR = 1.94, 95% CI: 1.20–3.15, *p* = 0.0070) and extended pad and phone usage (OR = 1.33, 95% CI: 1.01–1.77, *p* = 0.0457) were identified as risk factors for disfiguring dermatosis. Conversely, physical exercise (OR = 0.63, 95% CI: 0.41–0.97, *p* = 0.0358) and cosmetic usage (OR = 0.68, 95% CI: 0.48–0.95, *p* = 0.0241) were identified as protective factors against disfiguring dermatosis.

Regarding acne, the influencing factors were found similar to those observed for disfiguring dermatosis. Gender of male, warm water for washing face, frequent sun protection, frequent use of moisturizing products and extended use of pad and phone were identified as independent risk factors for acne, while physical exercise and cosmetic usage were identified as independent protective factors. Apart from the risk factors shared with disfiguring dermatosis, male participants exhibited a significantly higher risk of developing acne compared to female participants (OR = 6.62, 95% CI: 1.01–43.26, *p* = 0.0483). Moreover, participants who utilized a higher number of moisturizing products were associated with an increased risk of developing acne (OR = 1.86, 95% CI: 1.02–3.40, *p* = 0.0466).

Regarding melasma, the findings revealed that age, defecation regularity, frequency of physical exercise, frequency of sun protection and time of computer use were identified as independent and significant influencing factors. Apart from the frequency of exercise, the other factors were associated with an increased risk of melasma. Notably, defecation emerged as a distinct influencing factor for melasma, with irregular bowel movement significantly correlated with a higher risk of melasma (OR = 2.99, 95% CI: 1.11–8.08, *p* = 0.0303).

In the case of rosacea, individuals with a higher BMI were found to be at an increased risk of the condition (OR = 1.17, 95% CI: 1.01–1.35, *p* = 0.0357). Furthermore, participants who were unsure about the sensitivity of their skin exhibited a higher risk of rosacea compared to individuals with non‐sensitive skin (OR = 3.47, 95% CI: 1.03–11.68, *p* = 0.0441).

### 3.2 Dose–response relationship for facial disfiguring dermatosis, acne, melasma, and rosacea

**FIGURE 3:**
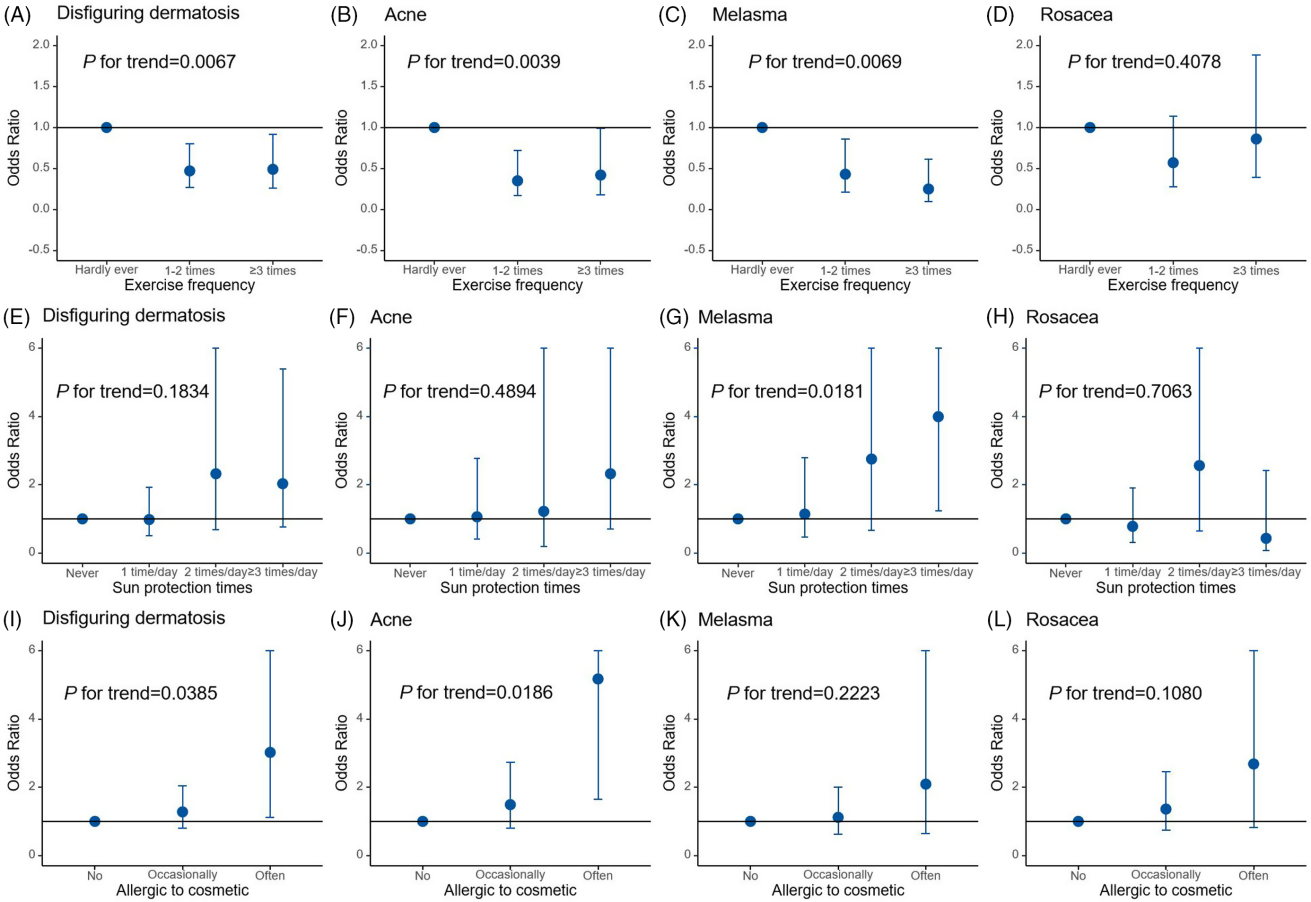
Effects of exercise frequency, sun protection times, and allergic to cosmetic on facial disfiguring dermatosis, acne, melisma, and rosacea. The results of associations from the univariate logistic regression, adjusting for sex, age and nation. Estimates are shown as odds ratios (ORs) and 95% CI. The risk factors were specified in both categories and linear variables. The category results are shown in the figure above, and the linear results are shown in Table 2. The *p‐*value for the trend is verified by the Cochran Armitage test.

Results based on categories analysis for disfiguring dermatosis (acne, melasma, and rosacea) indicated the presence of dose–response relationships for exercise, sun protection, and cosmetic allergies across various frequency ranges, with the risk increasing or decreasing in accordance with frequency (Figure 3). Regarding exercise frequency, engaging in exercise 1–2 times per week was associated with a 0.53‐fold decrease in the risk of disfiguring dermatosis (OR = 0.47, 95% CI: 0.27–0.80, *p* = 0.0054), while exercising more than 3 times per week was associated with a 0.51‐fold decrease in the risk of disfiguring dermatosis (OR = 0.49, 95% CI: 0.26–0.92, *p* = 0.0260), compared to no exercise (*P*trend = 0.0067). The trends observed for exercise frequency and its associations with acne and melasma were similar to those observed for disfiguring dermatosis (*P*trend for acne = 0.00339, *P*trend for melasma = 0.0069), whereas the association with rosacea did not exhibit a dose–response relationship (*P*trend = 0.4078). Concerning sun protection, although Figure 3 illustrates an increase in the risk of disfiguring dermatosis, acne, and melasma with an increasing times of sun protection used, only melasma demonstrated a significant dose–response relationship (*P*trend for acne = 0.0181). Notably, dose–response relationships were observed between cosmetic allergies and disfiguring dermatosis (*P*trend = 0.0385) as well as acne (*P*trend = 0.0186). Participants who reported frequent allergic reactions to cosmetics were associated with a higher risk of disfiguring dermatosis (OR = 3.02, 95% CI: 1.11–8.21, *p* = 0.0300) and acne (OR = 5.17, 95% CI: 1.64–16.31, *p* = 0.0050) compared to those who did not report allergies.

## 4 DISCUSSION

This case–control study, involving a total of 399 individuals from different regions in China, identified several factors that were independently associated with facial disfiguring skin diseases.

Exercise has been reported to have benefits for various diseases,^15^ such as cardiovascular diseases, tumors, and Alzheimer's disease.^16^, ^17^, ^18^ Our results showed that exercise was a protective factor for facial disfiguring dermatosis (OR: 0.63, 95% CI: 0.41–0.97), especially for acne (OR: 0.31, 95% CI: 0.13–0.77) and melasma (OR: 0.42, 95% CI: 0.22–0.80). A further dose–response analysis showed a dose‐dependent negative correlation between exercise and overall disfiguring dermatosis, acne and melasma. Excessive secretion of cortisol is known to cause acne and hyperpigmentation.^19^ A meta‐analysis reported that cortisol level in response to stress could be decreased by physical exercise interventions to a certain extend.^20^ Therefore, exercise may be beneficial for patients with emotional stress induced facial disfiguring dermatosis like acne and melasma. Meanwhile, we observed a negative but non‐significant association between exercise and rosacea in all analyses. This could be explained by prevalence‐incidence bias. Physical exercise usually leads to an increase in skin temperature and activation of sweat glands. So that patients with rosacea might refrain from participating in exercise for fear that the “flushing” facial condition may exacerbate.

With the advancement of science and technology, the use of computers and ipad has become increasingly prevalent. Meanwhile, the blue light emitted by the digital screens can do harm to human bodies when it comes to an extended usage. Our study revealed that extended use of ipad was a risk factor for acne (OR: 2.24, 95% CI: 1.25–4.02). Blue light exerts its harmful effect by interfering the circadian rhythm, producing reactive oxygen species in the skin and activating melanogenesis.^21^, ^22^ A previous study reported that individuals who spent less than 2 h per day using computers had a lower risk of acne compared to those who spent more time using computers,^23^ consistent with our findings. Furthermore, we found that extended computer use was a significant risk factor for melasma (OR: 1.59, 95%CI: 1.09–2.31). However, the specific mechanisms underlying these effects remain unclear and warrant further investigation.

In our study, a higher percentage of individuals in the melasma group reported irregular defecation compared to the control group (34.58% vs. 18.69%), indicating a strong association between irregular bowel movement and melasma (OR: 2.99; 95%CI: 1.11–8.08). This association may be attributed to gut microbiota dysbiosis, which is known to cause gastrointestinal symptoms like constipation and diarrhea.^24^, ^25^ Furthermore, studies have suggested that the dysregulated gut microbial composition of melasma patients is linked to the modulation of estrogen metabolism.^26^ The occurrence and development of melasma are closely related to abnormal estrogen metabolism,^27^ with β‐glucuronidase secreted by certain gut microbiota promoting the reabsorption of estrogen in the intestine and subsequently transporting it to other parts of the body, including the skin, via the circulatory system.^28^

Unlike acne and melasma, our study did not find any exogenous exposure factors associated with the occurrence of rosacea. Our data indicated that a higher BMI was associated with an increased risk of developing rosacea (OR: 1.17; 95%CI: 1.01–1.35). Although previous studies have yielded inconsistent findings on the relationship between rosacea and BMI, a recent cohort study in the United States women showed that BMI significantly increased the risk of rosacea in a dose‐dependent manner,^29^ consistent with our findings. Several mechanisms may underlie the link between obesity and rosacea, including a chronic and low‐grade inflammatory state and vascular function and structure changes.^30^, ^31^ However, the detailed pathophysiology needed further study to verify.

Our study had strengths in that we utilized an internet platform for questionnaire collection, achieving the goal of a multicenter case–control study, and providing a comparative analysis of acne, melasma, and rosacea, which can cause disfigurement. We further analyzed dose–response relationships for variables showing significant correlations, aiming to explore relevant risk and protective factors suitable for the Chinese population from multiple perspectives. We also verified some overlooked exposure factors, such as water temperature and exercise. In addition, we acknowledge that our study had limitations. Although our sample size was deemed valid based on the calculation of the minimum sample size,^32^ it was not sufficiently large, which may hide certain meaningful influencing factors. Furthermore, all individuals included in our study were Chinese population. The ethnic differences limited the extrapolation of our results. Finally, as our study focused on a population‐based approach, individual differences between people should be considered, and some factors related to individual susceptibility were not fully revealed through data analysis. Larger cohort research and large genetic databases are needed to deeply study and confirm.

## 5 CONCLUSION

Our study found that exercise was an independent protective factor for facial disfiguring dermatosis, while extended use of electronic equipment was an independent risk factor for acne and melasma. The occurrence of melasma was associated with irregular defecation. BMI might be the main reason for rosacea development. Our study provided new clues for the treatment and prevention of facial disfiguring dermatosis in the future.

## AUTHOR CONTRIBUTIONS

Qingyue Xia and Dan Luo were responsible for the conception and design of the study. Zhaopeng Wang, Qingyue Xia, Xingbao Luan, Lipan Fan, Xiaomei Cui, Hongjin Wu and Yuan Li collected the data. Yingdan Tang, Tianxurun Deng, Zhaopeng Wang and Qingyue Xia analyzed the data. Qingyue Xia wrote the original draft and Zhaopeng Wang helped to revise the draft. Dan Luo and Yang Zhao gave final approval of the version to be published. All authors have read and agreed to the published version of the manuscript.

## FUNDING INFORMATION

The study was financially supported by National Natural Science Foundation of China (81972961, 81771512).

## CONFLICT OF INTEREST STATEMENT

The authors have no conflict of interest to disclose.

## ETHICS STATEMENT

This study was approved by the medical ethics committee of Jiangsu Province Hospital, The First Affiliated Hospital of Nanjing Medical University (2023‐SR‐421).

## Supporting information


Table S1:

## Data Availability

The data that supports the findings of this study are available in the supplementary material of this article.
