# Supplementary material for: Exploring the influencing factors on acne, melasma, and rosacea: A case–control study in China
Source: J Cosmet Dermatol. 2024 Aug 2;23(12):4129–39. doi: 10.1111/jocd.16499 (PMC11626300; doi:10.1111/jocd.16499)
Supplement: Supplementary file 1 — Table S1: [file JOCD-23--s001.docx]

**Table S1. Baseline characters of Facial disfiguring dermatosis, Acne, Melasma, Rosacea and healthy controls.**

| **Baseline Characteristic** | **No disease**  **(N=107)** | **Disfiguring dermatosis**  **(N=292)** | **Acne**  **(N=94)** | **Melasma**  **(N=107)** | **Rosacea**  **(N=91)** |
| --- | --- | --- | --- | --- | --- |
| **Demographic and socioeconomic characteristics** |  |  |  |  |  |
| Sex |  |  |  |  |  |
| Female | 91(85.05) | 245(83.90) | 73(77.66) | 97(90.65) | 75(82.42) |
| Male | 16(14.95) | 47(16.10) | 21(22.34) | 10(9.35) | 16(17.58) |
| Age | 27.59(8.8) | 29.57(11.00) | 26.33(10.00) | 33.00(8.54) | 28.00(10.00) |
| Nation |  |  |  |  |  |
| Han nationality | 104(97.20) | 278(95.21) | 89(94.68) | 100(93.46) | 89(97.80) |
| Other | 3(2.80) | 14(4.79) | 5(5.32) | 7(6.54) | 2(2.20) |
| Marriage |  |  |  |  |  |
| Married | 53(49.53) | 138(47.26) | 35(37.23) | 64(59.81) | 39(42.86) |
| Unmarried | 53(49.53) | 142(48.63) | 56(59.57) | 37(34.58) | 49(53.85) |
| Divorce | 1(0.93) | 10(3.42) | 1(1.06) | 6(5.61) | 3(3.30) |
| Widowed | 0(0.00) | 2(0.68) | 2(2.13) | 0(0.00) | 0(0.00) |
| Family member |  |  |  |  |  |
| 1 | 1(0.93) | 4(1.37) | 0(0.00) | 3(2.80) | 1(1.10) |
| 2 | 5(4.67) | 22(7.53) | 11(11.70) | 5(4.67) | 6(6.59) |
| 3 | 30(28.04) | 80(27.40) | 29(30.85) | 29(27.10) | 22(24.18) |
| 4 | 33(30.84) | 78(26.71) | 23(24.47) | 26(24.30) | 29(31.87) |
| ≥5 | 38(35.51) | 108(36.99) | 31(32.98) | 44(41.12) | 33(36.26) |
| Education |  |  |  |  |  |
| Not attending primary school | 1(0.93) | 1(0.34) | 1(1.06) | 0(0.00) | 0(0.00) |
| Primary school | 0(0.00) | 8(2.74) | 1(1.06) | 6(5.61) | 1(1.10) |
| Junior high school | 11(10.28) | 30(10.27) | 7(7.45) | 16(14.95) | 7(7.69) |
| Senior high school | 14(13.08) | 33(11.30) | 8(8.51) | 19(17.76) | 6(6.59) |
| Technical school | 1(0.93) | 5(1.71) | 1(1.06) | 2(1.87) | 2(2.20) |
| Technical secondary school | 9(8.41) | 13(4.45) | 5(5.32) | 4(3.74) | 4(4.40) |
| Secondary vocational school | 3(2.80) | 4(1.37) | 1(1.06) | 2(1.87) | 1(1.10) |
| Junior college | 22(20.56) | 71(24.32) | 24(25.53) | 23(21.50) | 24(26.37) |
| Higher vocational education | 2(1.87) | 2(0.68) | 0(0.00) | 0(0.00) | 2(2.20) |
| Undergraduate | 40(37.38) | 108(36.99) | 44(46.81) | 29(27.10) | 35(38.46) |
| Postgraduate or above | 4(3.74) | 17(5.82) | 2(2.13) | 6(5.61) | 9(9.89) |
| BMI | 20.52(2.62) | 20.96(3.94) | 21.47(4.89) | 21.30(3.48) | 20.58(3.61) |
| Economy |  |  |  |  |  |
| ＜20000 | 26(24.30) | 88(30.14) | 29(30.85) | 31(28.97) | 28(30.77) |
| 20000~59999 | 23(21.50) | 50(17.12) | 16(17.02) | 21(19.63) | 13(14.29) |
| 60000~99999 | 14(13.08) | 44(15.07) | 15(15.96) | 16(14.95) | 13(14.29) |
| 100000~199999 | 24(22.43) | 50(17.12) | 16(17.02) | 19(17.76) | 15(16.48) |
| 200000~399999 | 9(8.41) | 38(13.01) | 11(11.70) | 13(12.15) | 14(15.38) |
| ≥400000 | 11(10.28) | 22(7.53) | 7(7.45) | 7(6.54) | 8(8.79) |
| **Living Habits** |  |  |  |  |  |
| Milk habit |  |  |  |  |  |
| No | 58(54.21) | 163(55.82) | 51(54.26) | 58(54.21) | 54(59.34) |
| Yes | 49(45.79) | 129(44.18) | 43(45.74) | 49(45.79) | 37(40.66) |
| Milk content |  |  |  |  |  |
| 0ml | 58(54.21) | 163(55.82) | 51(54.26) | 58(54.21) | 54(59.34) |
| ＜250ml | 27(25.23) | 82(28.08) | 25(26.60) | 31(28.97) | 26(28.57) |
| ≥250ml | 22(20.56) | 47(16.10) | 18(19.15) | 18(16.82) | 11(12.09) |
| Soft drink |  |  |  |  |  |
| Hardly ever | 70(65.42) | 198(67.81) | 66(70.21) | 70(65.42) | 62(68.13) |
| 1-2 times | 29(27.10) | 66(22.60) | 20(21.28) | 25(23.36) | 21(23.08) |
| ≥3 times | 8(7.48) | 28(9.59) | 8(8.51) | 12(11.21) | 8(8.79) |
| Milk tea |  |  |  |  |  |
| Hardly ever | 55(51.40) | 152(52.05) | 48(51.06) | 50(46.73) | 54(59.34) |
| 1-2 times | 45(42.06) | 116(39.73) | 37(39.36) | 47(43.93) | 32(35.16) |
| ≥3 times | 7(6.54) | 24(8.22) | 9(9.57) | 10(9.35) | 5(5.49) |
| Juice |  |  |  |  |  |
| Hardly ever | 65(60.75) | 161(55.14) | 51(54.26) | 58(54.21) | 52(57.14) |
| 1-2 times | 35(32.71) | 108(36.99) | 38(40.43) | 41(38.32) | 29(31.87) |
| ≥3 times | 7(6.54) | 23(7.88) | 5(5.32) | 8(7.48) | 10(10.99) |
| Diarrhea |  |  |  |  |  |
| Almost none | 75(70.09) | 193(66.10) | 64(68.09) | 71(66.36) | 58(63.74) |
| 1-3 times per week | 19(17.76) | 54(18.49) | 16(17.02) | 15(14.02) | 23(25.27) |
| 4-7 times per week | 2(1.87) | 11(3.77) | 4(4.26) | 5(4.67) | 2(2.20) |
| ≥1 time/day | 11(10.28) | 34(11.64) | 10(10.64) | 16(14.95) | 8(8.79) |
| Defecate times |  |  |  |  |  |
| 2 times/day | 21(24.14) | 42(19.53) | 13(18.31) | 16(22.86) | 13(17.57) |
| 1 time/day | 50(57.47) | 119(55.35) | 43(60.56) | 37(52.86) | 39(52.70) |
| 1 time/2 days | 10(11.49) | 37(17.21) | 11(15.49) | 8(11.43) | 18(24.32) |
| ≤1 time/3 days | 6(6.90) | 17(7.91) | 4(5.63) | 9(12.86) | 4(5.41) |
| Defecate irregular |  |  |  |  |  |
| No | 87(81.31) | 215(73.63) | 71(75.53) | 70(65.42) | 74(81.32) |
| Yes | 20(18.69) | 77(26.37) | 23(24.47) | 37(34.58) | 17(18.68) |
| Pet |  |  |  |  |  |
| No | 96(89.72) | 249(85.27) | 82(87.23) | 91(85.05) | 76(83.52) |
| Yes | 11(10.28) | 43(14.73) | 12(12.77) | 16(14.95) | 15(16.48) |
| Exercise frequency |  |  |  |  |  |
| Hardly ever | 56(52.34) | 202(69.18) | 70(74.47) | 76(71.03) | 56(61.54) |
| 1-2 times | 31(28.97) | 52(17.81) | 14(14.89) | 20(18.69) | 18(19.78) |
| ≥3 times | 20(18.69) | 38(13.01) | 10(10.64) | 11(10.28) | 17(18.68) |
| Sedentariness |  |  |  |  |  |
| Hardly ever | 6(5.61) | 15(5.14) | 2(2.13) | 10(9.35) | 3(3.30) |
| <1 hours | 6(5.61) | 14(4.79) | 5(5.32) | 6(5.61) | 3(3.30) |
| 1-2 hours | 19(17.76) | 43(14.73) | 21(22.34) | 13(12.15) | 9(9.89) |
| 3 -6 hours | 26(24.30) | 118(40.41) | 38(40.43) | 38(35.51) | 42(46.15) |
| ≥7 hours | 50(46.73) | 102(34.93) | 28(29.79) | 40(37.38) | 34(37.36) |
| **Genetic factor** |  |  |  |  |  |
| First-degree relatives |  |  |  |  |  |
| No | 72(67.29) | 196(67.12) | 57(60.64) | 75(70.09) | 64(70.33) |
| Yes | 35(32.71) | 96(32.88) | 37(39.36) | 32(29.91) | 27(29.67) |
| Second-degree relatives |  |  |  |  |  |
| No | 78(72.90) | 226(77.40) | 73(77.66) | 85(79.44) | 68(74.73) |
| Yes | 29(27.10) | 66(22.60) | 21(22.34) | 22(20.56) | 23(25.27) |
| Blood |  |  |  |  |  |
| Type A | 20(18.69) | 49(16.78) | 18(19.15) | 18(16.82) | 13(14.29) |
| Type B | 25(23.36) | 63(21.58) | 14(14.89) | 25(23.36) | 24(26.37) |
| Type O | 29(27.10) | 85(29.11) | 25(26.60) | 31(28.97) | 29(31.87) |
| Type AB | 7(6.54) | 19(6.51) | 4(4.26) | 9(8.41) | 6(6.59) |
| Don't know | 26(24.30) | 76(26.03) | 33(35.11) | 24(22.43) | 19(20.88) |
| **Environmental exposure** |  |  |  |  |  |
| Smoking |  |  |  |  |  |
| No | 98(91.59) | 262(89.73) | 83(88.30) | 99(92.52) | 80(87.91) |
| Yes | 9(8.41) | 30(10.27) | 11(11.70) | 8(7.48) | 11(12.09) |
| Smoking year |  |  |  |  |  |
| Non-smoking | 98(91.59) | 262(89.73) | 83(88.30) | 99(92.52) | 80(87.91) |
| < 1 years | 0(0.00) | 5(1.71) | 3(3.19) | 2(1.87) | 0(0.00) |
| < 3 years | 3(2.80) | 9(3.08) | 6(6.38) | 0(0.00) | 3(3.30) |
| ≥3 years | 6(5.61) | 16(5.48) | 2(2.13) | 6(5.61) | 8(8.79) |
| Smoking frequency |  |  |  |  |  |
| Non-smoking | 98(91.59) | 262(89.73) | 83(88.30) | 99(92.52) | 80(87.91) |
| 1 pack/2-multiple days | 7(6.54) | 23(7.88) | 10(10.64) | 4(3.74) | 9(9.89) |
| 1-multiple packs/day | 2(1.87) | 7(2.40) | 1(1.06) | 4(3.74) | 2(2.20) |
| Second smoking |  |  |  |  |  |
| Hardly ever | 65(60.75) | 157(53.77) | 51(54.26) | 55(51.40) | 51(56.04) |
| ＜5 | 30(28.04) | 96(32.88) | 29(30.85) | 34(31.78) | 33(36.26) |
| ＜10 | 7(6.54) | 25(8.56) | 9(9.57) | 11(10.28) | 5(5.49) |
| ≥10 | 5(4.67) | 14(4.79) | 5(5.32) | 7(6.54) | 2(2.20) |
| Drinking |  |  |  |  |  |
| No | 90(84.91) | 231(79.11) | 71(75.53) | 89(83.18) | 71(78.02) |
| Yes | 16(15.09) | 61(20.89) | 23(24.47) | 18(16.82) | 20(21.98) |
| Drinking year |  |  |  |  |  |
| Non-drinking | 91(85.05) | 231(79.11) | 71(75.53) | 89(83.18) | 71(78.02) |
| ＜1 years | 1(0.93) | 16(5.48) | 4(4.26) | 7(6.54) | 5(5.49) |
| ＜3 years | 7(6.54) | 13(4.45) | 10(10.64) | 0(0.00) | 3(3.30) |
| ＜5 years | 4(3.74) | 15(5.14) | 5(5.32) | 4(3.74) | 6(6.59) |
| ≥ 5 years | 4(3.74) | 17(5.82) | 4(4.26) | 7(6.54) | 6(6.59) |
| Drinking frequency |  |  |  |  |  |
| Non-drinking | 91(85.05) | 231(79.11) | 71(75.53) | 89(83.18) | 71(78.02) |
| Hardly ever | 4(3.74) | 26(8.90) | 10(10.64) | 8(7.48) | 8(8.79) |
| 1 time | 8(7.48) | 22(7.53) | 8(8.51) | 6(5.61) | 8(8.79) |
| 2-4 times | 3(2.80) | 12(4.11) | 4(4.26) | 4(3.74) | 4(4.40) |
| ≥5 times | 1(0.93) | 1(0.34) | 1(1.06) | 0(0.00) | 0(0.00) |
| Workplace |  |  |  |  |  |
| Outdoor | 3(2.80) | 23(7.88) | 6(6.38) | 11(10.28) | 6(6.59) |
| Indoor | 98(91.59) | 250(85.62) | 79(84.04) | 90(84.11) | 81(89.01) |
| Other | 6(5.61) | 19(6.51) | 9(9.57) | 6(5.61) | 4(4.40) |
| Sun shower |  |  |  |  |  |
| ＜2 hours | 90(84.11) | 232(79.45) | 75(79.79) | 84(78.50) | 73(80.22) |
| ＜4 hours | 13(12.15) | 38(13.01) | 14(14.89) | 12(11.21) | 12(13.19) |
| ＜6 hours | 1(0.93) | 13(4.45) | 3(3.19) | 6(5.61) | 4(4.40) |
| ≥ 6 hours | 3(2.80) | 9(3.08) | 2(2.13) | 5(4.67) | 2(2.20) |
| **Skin-related factors** |  |  |  |  |  |
| Skin color |  |  |  |  |  |
| White | 29(27.10) | 91(31.16) | 28(29.79) | 30(28.04) | 33(36.26) |
| Yellowish | 60(56.07) | 147(50.34) | 50(53.19) | 54(50.47) | 43(47.25) |
| Slightly black | 8(7.48) | 32(10.96) | 8(8.51) | 15(14.02) | 9(9.89) |
| Black | 4(3.74) | 5(1.71) | 1(1.06) | 2(1.87) | 2(2.20) |
| Don't know | 6(5.61) | 17(5.82) | 7(7.45) | 6(5.61) | 4(4.40) |
| Skin sensitive |  |  |  |  |  |
| No | 44(41.12) | 91(31.16) | 20(21.28) | 44(41.12) | 27(29.67) |
| Yes | 48(44.86) | 135(46.23) | 54(57.45) | 38(35.51) | 43(47.25) |
| Don't know | 15(14.02) | 66(22.60) | 20(21.28) | 25(23.36) | 21(23.08) |
| Wash water temperature |  |  |  |  |  |
| Cold | 52(48.60) | 103(35.27) | 28(29.79) | 43(40.19) | 32(35.16) |
| Warm | 53(49.53) | 178(60.96) | 60(63.83) | 62(57.94) | 56(61.54) |
| Hot | 2(1.87) | 11(3.77) | 6(6.38) | 2(1.87) | 3(3.30) |
| Wash face time |  |  |  |  |  |
| 1 time or less/day | 14(13.08) | 49(16.78) | 10(10.64) | 26(24.30) | 13(14.29) |
| 2 times/day | 84(78.50) | 224(76.71) | 80(85.11) | 71(66.36) | 73(80.22) |
| More than 3 times/day | 9(8.41) | 19(6.51) | 4(4.26) | 10(9.35) | 5(5.49) |
| Sun protection |  |  |  |  |  |
| Physical sunscreen | 35(32.71) | 79(27.05) | 35(37.23) | 24(22.43) | 20(21.98) |
| Sunscreen and other chemical sunscreen | 20(18.69) | 79(27.05) | 23(24.47) | 34(31.78) | 22(24.18) |
| Chemical+ Physical sunscreen | 32(29.91) | 77(26.37) | 18(19.15) | 30(28.04) | 29(31.87) |
| No sunscreen | 20(18.69) | 57(19.52) | 18(19.15) | 19(17.76) | 20(21.98) |
| Sun protection times |  |  |  |  |  |
| Never | 32(42.11) | 68(39.77) | 23(46.00) | 22(30.56) | 23(46.94) |
| 1 time/day | 33(43.42) | 60(35.09) | 16(32.00) | 27(37.50) | 17(34.69) |
| 2 time/day | 4(5.26) | 18(10.53) | 2(4.00) | 9(12.50) | 7(14.29) |
| 3 or more times/day | 7(9.21) | 25(14.62) | 9(18.00) | 14(19.44) | 2(4.08) |
| Moisturizing times |  |  |  |  |  |
| Never | 10(9.35) | 20(6.85) | 7(7.45) | 5(4.67) | 8(8.79) |
| Occasionally | 8(7.48) | 29(9.93) | 8(8.51) | 12(11.21) | 9(9.89) |
| 1 times/day | 17(15.89) | 56(19.18) | 14(14.89) | 24(22.43) | 18(19.78) |
| 2 times/day | 66(61.68) | 160(54.79) | 57(60.64) | 52(48.60) | 51(56.04) |
| 3 times/day | 4(3.74) | 10(3.42) | 2(2.13) | 6(5.61) | 2(2.20) |
| More than 3 times/day | 2(1.87) | 17(5.82) | 6(6.38) | 8(7.48) | 3(3.30) |
| Facial mask |  |  |  |  |  |
| Never | 14(13.08) | 37(12.67) | 15(15.96) | 11(10.28) | 11(12.09) |
| Occasionally | 55(51.40) | 148(50.68) | 52(55.32) | 51(47.66) | 45(49.45) |
| 2-6 times/week | 23(21.50) | 71(24.32) | 20(21.28) | 28(26.17) | 23(25.27) |
| 1 time/day | 12(11.21) | 19(6.51) | 4(4.26) | 9(8.41) | 6(6.59) |
| More than 2 times/day | 3(2.80) | 17(5.82) | 3(3.19) | 8(7.48) | 6(6.59) |
| Exfoliate |  |  |  |  |  |
| Never | 53(49.53) | 139(47.60) | 45(47.87) | 44(41.12) | 50(54.95) |
| Occasionally | 35(32.71) | 89(30.48) | 26(27.66) | 38(35.51) | 25(27.47) |
| 1time/2-3 weeks | 2(1.87) | 10(3.42) | 5(5.32) | 3(2.80) | 2(2.20) |
| 1time/week | 6(5.61) | 24(8.22) | 10(10.64) | 9(8.41) | 5(5.49) |
| 2-7 times/week | 4(3.74) | 16(5.48) | 5(5.32) | 5(4.67) | 6(6.59) |
| More than 1 times/day | 7(6.54) | 14(4.79) | 3(3.19) | 8(7.48) | 3(3.30) |
| Cosmetic |  |  |  |  |  |
| Never | 21(19.63) | 74(25.34) | 26(27.66) | 26(24.30) | 22(24.18) |
| Rarely | 30(28.04) | 79(27.05) | 29(30.85) | 27(25.23) | 23(25.27) |
| Occasionally | 31(28.97) | 92(31.51) | 25(26.60) | 34(31.78) | 33(36.26) |
| Often | 25(23.36) | 47(16.10) | 14(14.89) | 20(18.69) | 13(14.29) |
| Allergic to cosmetic |  |  |  |  |  |
| No | 53(49.53) | 120(41.10) | 36(38.30) | 47(43.93) | 37(40.66) |
| Occasionally | 49(45.79) | 139(47.60) | 44(46.81) | 50(46.73) | 45(49.45) |
| Often | 5(4.67) | 33(11.30) | 14(14.89) | 10(9.35) | 9(9.89) |
| **Use of electronic equipment** |  |  |  |  |  |
| Computer hours |  |  |  |  |  |
| ≤2 hours | 46(42.99) | 109(37.33) | 39(41.49) | 34(31.78) | 36(39.56) |
| ＜4 hours | 15(14.02) | 38(13.01) | 14(14.89) | 14(13.08) | 10(10.99) |
| ＜6 hours | 6(5.61) | 40(13.70) | 9(9.57) | 15(14.02) | 16(17.58) |
| ≥6 hours | 40(37.38) | 105(35.96) | 32(34.04) | 44(41.12) | 29(31.87) |
| Pad phone hours |  |  |  |  |  |
| ≤2 hours | 16(14.95) | 50(17.12) | 11(11.70) | 22(20.56) | 17(18.68) |
| ＜4 hours | 32(29.91) | 46(15.75) | 15(15.96) | 17(15.89) | 14(15.38) |
| ＜6 hours | 15(14.02) | 56(19.18) | 15(15.96) | 24(22.43) | 17(18.68) |
| ≥6 hours | 44(41.12) | 140(47.95) | 53(56.38) | 44(41.12) | 43(47.25) |
| **Influence on daily life** |  |  |  |  |  |
| Influence |  |  |  |  |  |
| No influence | 25(23.36) | 69(23.63) | 15(15.96) | 36(33.64) | 18(19.78) |
| Some influence | 59(55.14) | 157(53.77) | 43(45.74) | 60(56.07) | 54(59.34) |
| Large influence | 15(14.02) | 49(16.78) | 27(28.72) | 10(9.35) | 12(13.19) |
| Very large influence | 8(7.48) | 17(5.82) | 9(9.57) | 1(0.93) | 7(7.69) |

BMI, body mass index.

**Table S2. Association of Risk factors with Facial disfiguring dermatosis by Multivariate logistic regression.**

| **Variables** | **Disfiguring dermatosis** | |
| --- | --- | --- |
|  | **OR (95%CI)** | ***P* value** |
| **Demographic and socioeconomic** |  |  |
| sex | 1.19(0.45, 3.16) | 0.7260 |
| age | 1.06(1.01, 1.11) | 0.0276 |
| nation | 0.81(0.13, 4.95) | 0.8227 |
| BMI | 1.07(0.98, 1.18) | 0.1313 |
| **Living Habits** |  |  |
| Defecate irregular | 1.69(0.81, 3.53) | 0.1597 |
| Exercise frequency | 0.63(0.41, 0.97) | 0.0358 |
| **Environmental exposure** |  |  |
| Workplace |  |  |
| Outdoor | ref |  |
| Indoor | 0.28(0.07, 1.22) | 0.0899 |
| Other | 0.18(0.03, 1.19) | 0.0755 |
| **Skin-related factors** |  |  |
| Sun protection times | 1.94(1.20, 3.15) | 0.0070 |
| Cosmetic | 0.68(0.48, 0.95) | 0.0241 |
| Allergic to cosmetic | 1.13(0.63, 2.03) | 0.6779 |
| Skin color |  |  |
| White | ref |  |
| Yellowish | 0.61(0.29, 1.28) | 0.1881 |
| Slightly black | 1.03(0.30, 3.55) | 0.9599 |
| Black | 0.23(0.04, 1.27) | 0.0918 |
| Don't know | 0.64(0.11, 3.86) | 0.6267 |
| Skin sensitive |  |  |
| No | ref |  |
| Yes | 0.86(0.41, 1.80) | 0.6863 |
| Don't know | 1.44(0.57, 3.65) | 0.4367 |
| Wash water temperature |  |  |
| Cold | ref |  |
| Warm | 3.00(1.51, 5.95) | 0.0016 |
| Hot | 5.62(0.97, 32.48) | 0.0535 |
| Sun protection |  |  |
| Physical sunscreen | ref |  |
| Sunscreen and other chemical sunscreen | 1.10(0.41, 2.96) | 0.8554 |
| Chemical+ Physical sunscreen | 0.60(0.23, 1.51) | 0.2763 |
| No sunscreen | 1.50(0.56, 3.98) | 0.4159 |
| **Use of electronic equipment** |  |  |
| Pad phone hours | 1.33(1.01, 1.77) | 0.0457 |

The results of associations from the multivariate logistic regression. Estimates are odds ratios (OR) and 95%CI. The multi-variate regression model included all risk factors with *P*-value less than 0.2 obtained by univariate regression, adjusting for age, sex, and nation.

**Table S3. Association of Risk factors with Acne by Multivariate logistic regression.**

| **Variables** | **Acne** | |
| --- | --- | --- |
|  | **OR (95%CI)** | ***P* value** |
| **Demographic and socioeconomic** |  |  |
| Sex | 6.62(1.01, 43.26) | 0.0483 |
| Age | 1.04(0.96, 1.12) | 0.3519 |
| Nation | 1.24(0.07, 21.51) | 0.8820 |
| Family member | 1.18(0.70, 1.99) | 0.5350 |
| Education | 1.05(0.84, 1.29) | 0.6859 |
| BMI | 1.13(1.00, 1.28) | 0.0581 |
| **Living Habits** |  |  |
| Exercise frequency | 0.31(0.13, 0.77) | 0.0112 |
| **Environmental exposure** |  |  |
| Drinking | 0.89(0.24, 3.33) | 0.8667 |
| **Skin-related factors** |  |  |
| Sun protection times | 2.66(1.17, 6.05) | 0.0196 |
| Moisturizing times | 1.86(1.02, 3.40) | 0.0446 |
| Cosmetic | 0.51(0.27, 0.97) | 0.0396 |
| Allergic to cosmetic | 1.82(0.70, 4.77) | 0.2210 |
| Skin color |  |  |
| White | ref |  |
| Yellowish | 8.26(0.57,119.37) | 0.1212 |
| Slightly black | 4.64(0.36, 59.06) | 0.2374 |
| Black | 5.95(0.27,130.20) | 0.2571 |
| Don't know | 0.69(0.02, 31.10) | 0.8464 |
| Skin sensitive |  |  |
| No | ref |  |
| Yes | 0.30(0.06, 1.41) | 0.1280 |
| Don't know | 1.51(0.34, 6.83) | 0.5895 |
| Wash water temperature |  |  |
| Cold | ref |  |
| Warm | 5.16(1.44, 18.54) | 0.0120 |
| Hot | 12.56(0.96,164.72) | 0.0540 |
| Sun protection |  |  |
| Physical sunscreen | ref |  |
| Sunscreen and other chemical sunscreen | 0.60(0.10, 3.56) | 0.5712 |
| Chemical+ Physical sunscreen | 0.32(0.06, 1.72) | 0.1839 |
| No sunscreen | 1.04(0.18, 5.94) | 0.9690 |
| **Use of electronic equipment** |  |  |
| Pad phone hours | 2.24(1.25, 4.02) | 0.0068 |
| **Influence on daily life** |  |  |
| Influence | 1.26(0.68, 2.36) | 0.4628 |

The results of associations from the multivariate logistic regression. Estimates are odds ratios (OR) and 95%CI. The multi-variate logistics model included all risk factors with *P*-value less than 0.2 obtained by univariate regression, adjusting for age, sex, and nation.

**Table S4. Association of Risk factors with Melasma by Multivariate logistic regression.**

| **Variables** | **Melasma** | |
| --- | --- | --- |
|  | **OR (95%CI)** | ***P* value** |
| **Demographic and socioeconomic** |  |  |
| Sex | 1.16(0.24, 5.60) | 0.8524 |
| Age | 1.15(1.07, 1.24) | 0.0003 |
| Nation | 3.22(0.25, 41.02) | 0.3673 |
| Marriage |  |  |
| Married | ref |  |
| Unmarried | 1.30(0.41, 4.07) | 0.6531 |
| Divorce | 7.09(0.49,103.13) | 0.1514 |
| Education | 1.12(0.94, 1.34) | 0.2074 |
| BMI | 1.09(0.96, 1.23) | 0.1689 |
| Economy | 0.92(0.70, 1.22) | 0.5653 |
| **Living Habits** |  |  |
| Milk tea | 1.06(0.49, 2.28) | 0.8771 |
| Irregular defecation | 2.99(1.11, 8.08) | 0.0303 |
| Exercise frequency | 0.42(0.22, 0.80) | 0.0088 |
| **Environmental exposure** |  |  |
| Second smoking | 0.90(0.51, 1.57) | 0.7010 |
| Workplace |  |  |
| Outdoor | ref |  |
| Indoor | 0.16(0.02, 1.25) | 0.0804 |
| Other | 0.25(0.02, 3.67) | 0.3097 |
| **Skin-related factors** |  |  |
| Wash face time | 0.45(0.18, 1.10) | 0.0785 |
| Sun protection times | 2.58(1.38, 4.82) | 0.0030 |
| Sun protection |  |  |
| Physical sunscreen | ref |  |
| Sunscreen and other chemical sunscreen | 1.75(0.50, 6.14) | 0.3835 |
| Chemical+ Physical sunscreen | 0.59(0.17, 2.03) | 0.4014 |
| No sunscreen | 1.72(0.44, 6.78) | 0.4372 |
| **Use of electronic equipment** |  |  |
| Computer hours | 1.59(1.09, 2.31) | 0.0152 |
| **Influence on daily life** |  |  |
| Influence | 0.86(0.47, 1.59) | 0.6410 |

The results of associations from the multivariate logistic regression. Estimates are odds ratios (OR) and 95%CI. The multi-variate logistic model included all risk factors with *P*-value less than 0.2 obtained by univariate regression, adjusting for age, sex, and nation.

**Table S5 Association of Risk factors with Rosacea by Multivariate logistic regression.**

| **Variables** | **Rosacea** | |
| --- | --- | --- |
|  | **OR (95%CI)** | ***P* value** |
| **Demographic and socioeconomic** |  |  |
| Sex | 1.65(0.48, 5.64) | 0.4217 |
| Age | 1.05(0.99, 1.11) | 0.1384 |
| Nation | 0.70(0.06, 7.84) | 0.7742 |
| Education | 1.19(0.96, 1.46) | 0.1055 |
| BMI | 1.17(1.01, 1.35) | 0.0357 |
| **Skin-related factors** |  |  |
| Allergic to cosmetic | 1.92(0.90, 4.12) | 0.0925 |
| Skin color |  |  |
| White |  |  |
| Yellowish | 0.79(0.30, 2.12) | 0.6435 |
| Slightly black | 0.78(0.16, 3.84) | 0.7581 |
| Black | 0.40(0.04, 4.01) | 0.4374 |
| Don't know | 0.15(0.02, 1.21) | 0.0746 |
| Skin sensitive |  |  |
| No | ref |  |
| Yes | 1.89(0.63, 5.63) | 0.2557 |
| Don't know | 3.47(1.03, 11.68) | 0.0441 |
| Wash water temperature |  |  |
| Cold | ref |  |
| Warm | 1.98(0.80, 4.95) | 0.1413 |
| Hot | 0.00(0.00, I ) | 0.9926 |
| Sun protection |  |  |
| Physical sunscreen | ref |  |
| Sunscreen and other chemical sunscreen | 3.74(0.88, 15.96) | 0.0749 |
| Chemical+ Physical sunscreen | 2.81(0.74, 10.70) | 0.1293 |
| No sunscreen | 2.33(0.55, 9.89) | 0.2495 |

The results of associations from the multivariate logistic regression. Estimates are odds ratios (OR) and 95%CI. The multivariate logistic model included all risk factors with *P*-value less than 0.2 obtained by univariate regression, adjusting for age, sex, and nation.
